# Instrumental assessment of pressure pain threshold over trigeminal and extra-trigeminal area in people with episodic and chronic migraine: a cross-sectional observational study

**DOI:** 10.1007/s10072-024-07372-4

**Published:** 2024-02-24

**Authors:** Manuela Deodato, Antonio Granato, Miriam Martini, Raffaele Sabot, Alex Buoite Stella, Paolo Manganotti

**Affiliations:** 1 Department of Medical, Surgical and Health Sciences, University of Trieste, Via Pascoli 31, 34100 Trieste, Italy; 2 Azienda Sanitaria Universitaria Giuliano Isontina, Via Pascoli 31, 34100 Trieste, Italy

**Keywords:** Migraine, Pressure pain threshold, Sensitization, Widespread pain, Hyperalgesia, Trigemino-cervical complex

## Abstract

**Background:**

Central and peripheral sensitization are characterized by widespread hyperalgesia that is manifested by larger pain extent area and reduction in pressure pain threshold (PPT). PPT decreases in patients with migraine not only over the trigeminal cervical complex but also throughout the body.

**Methods:**

A cross-sectional study was adopted to assess the local and widespread hyperalgesia in chronic and episodic migraine patients respect to healthy controls. The guidelines of Andersen’s were used to evaluate the PPT bilaterally over 3 muscles in the trigemino-cervical complex (temporalis, sub-occipitalis, trapezius) and over 1 muscle far from this area (tensor fasciae latae).

**Results:**

Thirty subjects with episodic migraine (35.8 ± 2.82 years), 30 with chronic migraine (53.03 ± 19.79 years), and 30 healthy controls (29.06 ± 14.03 years) were enrolled. The interaction effect was present for the trapezius muscle with a significant difference between the right and the left side in episodic group (*p* = 0.003). A group effect was highlighted in all four muscles analyzed such as suboccipital (*p* < 0.001), temporalis (*p* > 0.001), trapezius (*p* < 0.001), and TFL (*p* < 0.001). PPT was usually higher in the control group than in the episodic group which in turn was characterized by higher PPT values than the chronic group.

**Conclusions:**

People with chronic and episodic migraine presented lower PPT than healthy controls both in the trigeminal and in the extra-trigeminal area. People with chronic migraine presented lower PPT than episodic migraine only in the trigeminal area. Temporalis and sub-occipitalis are the most sensitive muscles in people with chronic and episodic migraine.

## Introduction

Headache is a common pain condition and a prevalent neurological disorder that is characterized by adverse cognitive, behavioral, and physical effects. Among primary headache, migraine causes a major burden on healthcare systems due to the high level of disability and comorbidity related to migraine [1, 2]. Furthermore, migraine involves a complex interplay between central and peripheral neuronal structures that complicate the understanding of the clinical manifestations and, consequently, the management of the patients [3].

The trigemino-cervical complex has a pivotal role in migraine pathophysiology [4–6]. Extensive research has shown that peripheral structures innervated by the trigemino-cervical complex and by the upper cervical spine may generate and maintain nociceptive afferents which, in turn, may be responsible for peripheral sensitization [7, 8]. As a consequence, peripheral sensitization could lead to an increase in the stimulation to the brain stem, hypothalamus, thalamus, and cortex which, in turn, could lead to central sensitization [9, 10]. Central and peripheral sensitizations are characterized by widespread pressure hyperalgesia that is manifested by larger pain extent area, amplification of pain, and reduction in pressure pain threshold (PPT). PPT reflects the sensitivity of soft tissue: a general lower PPT is commonly associated with central sensitization; a local lower PPT is commonly associated with peripheral sensitization [4].

Concerning migraine, it seems that PPT decreases in people with migraine compared to healthy controls, not only over trigemino-cervical complex but also throughout the body [11]. People with episodic and chronic migraine show several musculoskeletal dysfunctions compared to healthy controls, not only in the trigemino-cervical complex but throughout all spine: neck and low back pain; reduction in cervical and dorsal range of motion; forward head posture; pericranial tenderness; active trigger points [7, 12–15]. It is now well established that widespread pressure hyperalgesia is related to central sensitization or to the cycles of migraine attacks. On one side, migraine cycle changes the perception of pain: the ictal phase presents a lower pressure pain threshold than the interictal phase. This suggests that migraine attack generally increases pain intensity perception in the chronic as well as in the episodic form and in the symptomatic side as well as in the non-symptomatic side [11, 16–19]. On the other side, central sensitization changes the perception of pain: the alteration in excitatory-inhibitory modulation presents a larger pain extent area than normal condition; a larger pain extent area is linked to high disability and migraine chronification [3, 4]. This suggests that central sensitization increases generally pain intensity perception in the ictal as well as in the interictal phase of a migraine cycle.

The Andersen guideline has provided a method to measure the PPT in people with migraine [19]. No previous study has investigated the differences in PPT in people with chronic and episodic migraine respect to healthy controls, according to this guideline. No previous study has investigated the PPT in people with chronic and episodic migraine, in particular in the interictal phase without prophylactic or symptomatic medication, according to this guideline. Therefore, the aim of present study is to assess the differences in PPT over the trigemino-cervical and extra-trigemino-cervical areas in people with chronic and episodic migraine with respect to healthy controls, according to the Andersen guideline.

## Materials and methods

A cross-sectional study was adopted to assess the local and widespread hyperalgesia in people with chronic and episodic migraine. The study was carried out by the physiotherapy degree course and the neurological clinic, University of Trieste. The research was conducted in accordance with the Code of Ethics of the World Medical Association (Declaration of Helsinki) and it was approved by the institutional review board. All participants signed the informed consent.

The first evaluation was performed by two expert neurologists. Participants were selected by the following criteria of inclusion: diagnosis of chronic migraine by criteria of ICDH3-beta [20]; diagnosis of episodic migraine by criteria of ICDH3-beta [20]; age over 18. Conversely, the criteria of exclusion were as follows: pregnancy; serious psychiatric pathologies; serious pathologies such as traumas, tumors, or infections; significant surgical procedures during the previous 12 months; non-pharmacological or pharmacological prophylactic treatment in the previous three months. Next, a headache diary was given to each participant to record the headache parameters such as frequency (headache days a month), duration, and intensity of attacks. The headache diaries were re-evaluated after 1 month (baseline period). The eligibility criteria for chronic migraine required individuals to have headache frequency greater than or equal to 15 days per month and at least 50% of the headache’s days had to be characterized by migraine crisis (≥ 4 h continuous severe headache or one hour of headache with intake of symptomatic drugs). While the eligibility criteria for episodic migraine required to have headache frequency greater than or equal to five attacks per month (headache attacks lasting 4–72 h). As regards the healthy control groups, they were selected from teachers, students, and administrative staff of our University. The inclusion criteria were as follows: age from 18 to 70 years; no migraine, tension-type headache, or another primary headache form. The exclusion criteria were headache diagnosis and the same exclusion criteria for patients with chronic and episodic migraine. After, the pressure pain threshold (PPT) was assessed by one expert physiotherapist.

### Pressure pain threshold (PPT)

Data were collected using the algometer Somedic. Previous research has established the reliability and validity of the Algometer Sometic to assess the PPT in craniofacial muscles and sensitive areas due to the small surface [19, 21–25]. The guideline of Andersen [19] was adopted to evaluate the PPT bilaterally over 3 muscles in the trigemino-cervical complex (temporalis, sub-occipitalis, trapezius) and over one muscle far from this area (tensor fasciae latae). Prior to data collection, the patients received an explanation of the evaluation. The first pressure was applied on the wrist, patients were asked to press the stop button of the algometer when the pressure resulted in pain. Then, each muscle was assessed with three consecutive measures with an interval of 1 min. The pressure was applied with an increase in rate of 30 kPa/s. The same order of measurements was respected: first, temporalis right and tensor fascia latae right were assessed in right lateral decubitus; second, temporalis left and tensor fascia latae left were assessed in left lateral decubitus; third, sub-occipitalis right and trapezius right, and then sub-occipitalis left, trapezius left were assessed in prone position. In accordance with the guideline, for migraine participants, the measurements were conducted in the 3 days after the last migraine attack (pain-free period) [16–19]. All participants were asked to not take symptomatic medication, in addition the female’s participants were examined in the late follicular phase (from the next day after the end of menstruation to the day before ovulation) [19, 25, 26].

### Statistical analysis

The statistical analysis was performed with SPSS v.23 (IBM inc.). The Shapiro–Wilk test for normality of distribution was performed. A mixed-factors ANOVA was performed to test within factor (2 × side, right and left) and between factor (3 × groups: episodic, chronic, and healthy). In case of significant main effects, Bonferroni post hoc comparisons were performed and partial eta-squared (pη^2^) was used for effect size. Significance was set at *p* < 0.05.

The data were represented with the software GraphPad Prism 8.4.1.

## Results

A total of 90 subjects were enrolled, 30 with episodic migraine, 30 with chronic migraine, and 30 healthy controls. The episodic group consisted of 14 women and 16 men, mean age of 35.8 (SD ± 2.82); the chronic group consisted of 15 women and 15 men, mean age of 53.03 (SD ± 19.79) and the control group, similar in epidemiological characteristics to the other two groups, consisted of 13 women and 17 men, mean age of 29.06 (SD ± 14.03). As regard to frequency of headache attack per month, the chronic group presented 21.4 (SD ± 4) days, while the episodic group presented 10.5 (SD ± 3.3) days. Concerning the duration of illness chronic groups suffered from migraine for 15 (SD ± 11) years, while the episodic group suffered from migraine for 10 (SD ± 8.5) years.

### PPT

Comparing the PPT values of the three groups did not highlight the presence of a side*group effect in the sub-occipital (*F*_2,87_ = 2.537, *p* = 0.085, pη2 = 0.055), temporalis (*F*_2,87_ = 1.185, *p* = 0.311, pη2 = 0.027), and TFL (F_2,87_ = 0.360, *p* = 0.699, pη2 = 0.008) muscles. The interaction effect was instead present for the trapezius muscle (*F*_2,87_ = 4.108, *p* = 0.020, pη2 = 0.086), where a significant difference was highlighted between the right and left side in the episodic group (*p* = 0.003), but not in the chronic (*p* = 0.775) and control groups (*p* = 0.562). Furthermore, the episodic group presented higher values of PPT than chronic one on both sides, in particular 181.56 (95% CI: 83.61–279.50) kPa on the right side (*p* < 0.001) and 110.72 (95% CI: 0.302–221.13) kPa on the left one (*p* = 0.049). Nevertheless, the control group had higher values of PPT bilaterally than chronic (*p* < 0.001) and episodic (*p* < 0.001) groups.

A group effect was highlighted in all four muscles analyzed, such as suboccipital (*F*_2,87_ = 22.927, *p* < 0.001, pη2 = 0.345), temporalis (*F*_2,87_ = 28.332, *p* > 0.001, pη2 = 0.394), trapezius (*F*_2,87_ = 42.616, *p* < 0.001, pη2 = 0.495), and TFL (*F*_2,87_ = 19.758, *p* < 0.001, pη2 = 0.312).

The values of PPT in the episodic group were higher than chronic one, in particular for the suboccipitalis muscle the difference was 121.27 (95% IC: 5.70–236.83) kPa (*p* = 0.036), for the temporalis muscle 87.69 (95%IC: 16.97–158.41) kPa (*p* = 0.01) and for the trapezius muscle 146.14 (95% IC: 23.25–269.02) kPa (*p* = 0.014). Instead, there was not a significant difference for the TFL muscle (*p* = 0.746).

**Fig. 1:**
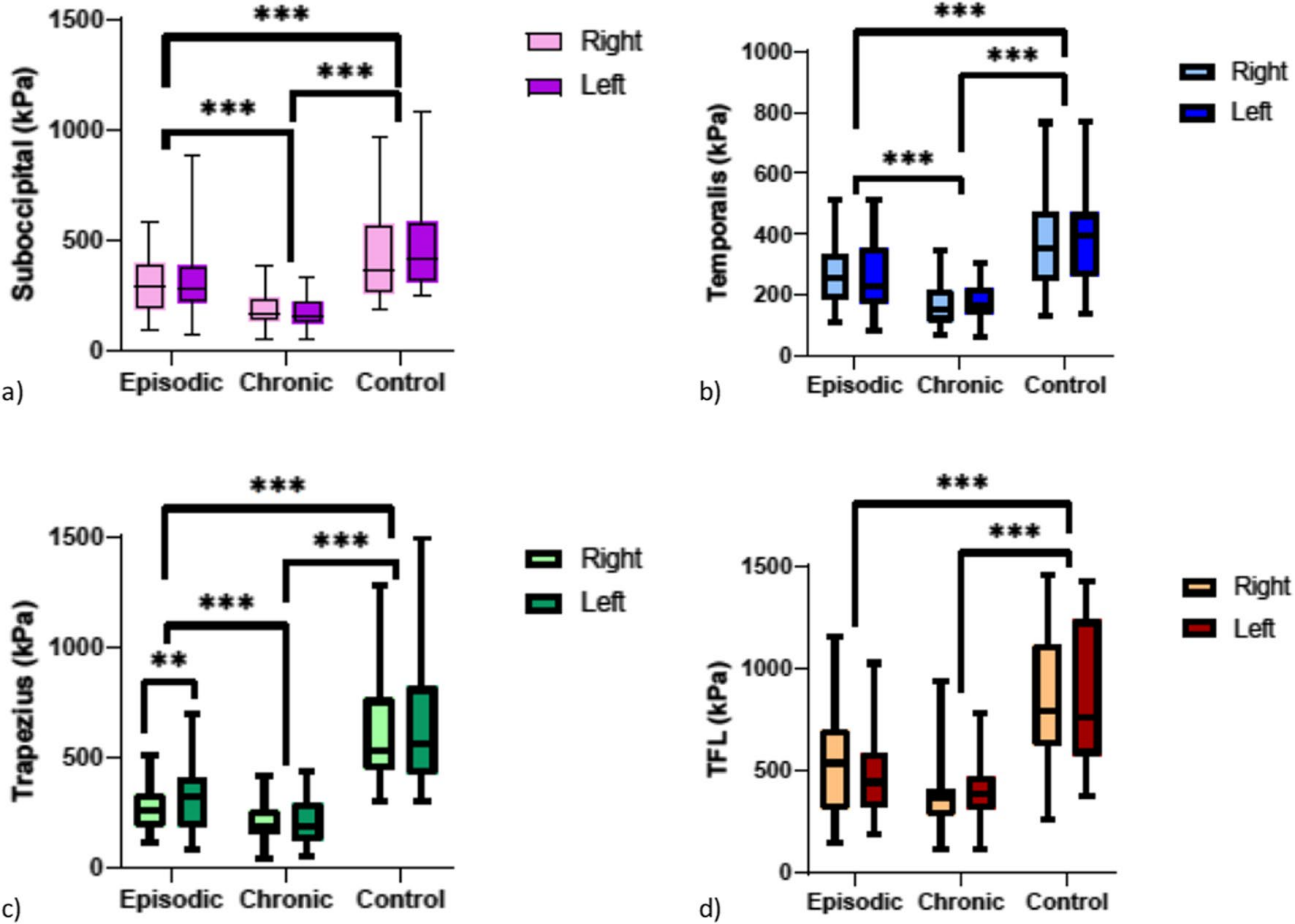
Pressure pain threshold (PPT) values of **a** suboccipital, **b** temporalis, **c** trapezius, and **d** tensor fasciae latae (TFL) muscles in episodic (*n* = 30), chronic (*n* = 30), and control (*n* = 30) groups. *p < 0.05; **p < 0.01; *p* < 0.001***. Post hoc significance values for side and group main effects at the mixed-factors ANOVA

Furthermore, the healthy group showed a significantly higher PPT in all the muscles analyzed both in comparison to episodic and chronic groups. In particular, in comparison with the episodic group, the PPT was 196.35 (95% CI: 80.78–311.91) kPa higher in the suboccipital muscle (*p* < 0.001), 129.06 (95% CI: 58.34–199.78) kPa in the temporalis (*p* < 0.001), 308.995 (95% CI: 186.10–431.883) kPa in Trapezius and (*p* < 0.001), and 508.82 (95% CI: 248.39–769.25) kPa in the TFL (*p* < 0.001). Then, in comparison with the chronic group, the PPT was 317.62 (95% CI: 202.05–433.18) kPa higher in the suboccipital muscle (*p* < 0.001), 216.76 (95% CI: 146.04–287.48) kPa in the temporalis (*p* < 0.001), 455.13 (95% CI: 332.24–578.02) kPa in Trapezius, and (*p* < 0.001) and 632.74 (95% CI: 372.31–893.170) kPa in the TFL (*p* < 0.001) (Table 1) (Fig. 1a, b, c, and d).

**Table 1:** Pressure pain threshold over trigeminal and extra-trigeminal area in people with episodic and chronic migraine and in healthy control

PPT (kPa)	Episodic *n* = 30	Chronic *n* = 30	Control *n* = 30	*p*-value interaction side*group	Group effect
Suboccipital				*p* = 0.085	*p* < 0.001
Right	290.07 (± 61.92)	193.00 (± 5.09)	439.98 (± 220.38)		
Left	300.11 (± 85.48)	153.50 (± 0.84)	559.53 (± 434.46)		
Temporalis				*p* = 0.311	*p* < 0.001
Right	268.22 (± 100.69)	194.30 (± 78.06)	385.46 (± 161.25)		
Left	250.04 (± 2.33)	161.00 (± 9.75)	399.75 (± 169.34)		
Trapezius				*p* = 0.020	*p* < 0.001
Right	383.31 (± 173.64)	232.85 (± 14.35)	659.68 (± 284.45)		
Left	318.53 (± 24.04)	207.4 (± 21.77)	675.14 (± 328.70)		
TFL				*p* = 0.699	*p* < 0.001
Right	528.04 (± 33.23)	371.15 (± 36.13)	1079.71 (± 1146.85)		
Left	467.80 (± 26.77)	365.85 (± 55.36)	942.67 (± 480.74)		

Bold values for *p* < 0.05 at the mixed-factors ANOVA side*group interaction effect and group simple main effect

## Discussion

Several studies have highlighted the great value of pressure pain threshold (PPT) assessment for clinicians and researchers to better understand pain mechanisms in patients with headaches [11, 19, 21–23]. Our study has highlighted the differences in the PPT among several muscles over the trigemino-cervical and extra-trigemino-cervical area in patients with episodic and chronic migraine, respect to healthy controls. One interesting finding was that healthy controls presented a higher value of PPT respect than episodic and chronic migraine people both in the trigeminal and extra-trigeminal area. Another important finding was that people with episodic migraine presented higher PPT than chronic migraine only in the trigeminal area but not in the extra-trigeminal area. Finally, the most sensitive muscles seem to be sub-occipitalis and temporalis.

The evaluation of pressure pain threshold (PPT) consists of a neurophysiological quantification of mechanical sensitivity of the muscles. In our study, people with episodic and chronic migraine presented lower PPT than healthy controls both over trigeminal and extra-trigeminal area. This confirms that the pathophysiology of migraine could change pain perception both in episodic and in chronic form [3, 4, 16]. In fact, the pathophysiology of migraine depends on two opposing processes, lack of habituation and sensitization, that, together, lead to an increased response to sensory stimulation [27] not only during a migraine attack (ictal phase), but especially between attacks (interictal phase). Sensitization in migraine is enhanced concerning habituation, especially during the migraine attack (ictal phase). Sensitization leads to an augmentation of central sensory signaling after noxious stimulation [3–6]: nociceptive input from peripheral structures could result in peripheral sensitization, which, in turn, could sensitize the first-order or second-order trigemino-vascular nociceptors. The consequent augmentation of the stimulation to the thalamus and cortex could reduce the physiological descending inhibitory painful transmission, which, in turn, could manifest itself clinically and neurophysiologically with hyperalgesia and allodynia [7, 8, 23].

Concerning the differences between chronic and episodic migraine, it seems that chronic migraine presented more local hyperalgesia in the trigeminal cervical area than widespread hyperalgesia respect to episodic migraine. Indeed, chronic migraine highlighted a lower PPT than episodic migraine only in the trigeminal area but not in the extra-trigeminal area: the differences in local hyperalgesia between chronic and episodic migraine in the trigeminal area may be related to the current migraine attack in the chronic form that could sensitize the trigemino-cervical complex; the similar widespread hyperalgesia between chronic and episodic migraine in the extra-trigeminal area may be related to central sensitization. These results are in line with previous studies concerning only the widespread hyperalgesia in chronic and episodic form [15, 27, 28] but not in line regarding the local hyperalgesia [15, 28, 29]. However, previous studies evaluated only the temporalis muscles in the trigeminal area [15, 28] and no study carried out the evaluation according to the Andersen guideline [19]. According to these data, we can infer that even though the chronicity could lead to central sensitization and in a larger pain extent area, people with chronic migraine present more local hyperalgesia in the trigemino-cervical complex rather than widespread hyperalgesia. A possible explanation for these results may be the trigeminal system pain pathway. The trigeminal system is at the center of the complex interplay between central and peripheral structures: peripheral and central afferent projections; direct and indirect ascending/descending projections. The nociceptive afferent projections from intra and extra cranial blood vessels and upper cervical spinal cord are relayed to many subcortical and cortical structures through the direct and indirect afferent ascending projections from trigemino-cervical complex. This nociceptive information is modulated by direct and indirect descending projections arising in the cortex: direct projections arise from primary somatosensory cortex, insular cortex, and hypothalamus to trigemino-cervical complex; indirect projections arise from primary somatosensory cortex through hypothalamus and from hypothalamus through locus coeruleus, periaqueductal gray and the rostral ventromedial medulla to trigemino-cervical complex [27]. As a consequence, the trigemino-cervical complex is subject to this complex network; this is why it plays a pivotal role in migraine transmission.

Concerning the differences among muscles, despite no statistical differences were found among muscles, the sub-occipitalis and the temporalis muscles seem to be the most sensitive muscles. This result further supports the idea that temporalis and sub-occipitalis are linked to headache due to their anatomical connections: temporalis is innervated by the trigeminal nerve; sub-occipitalis is innervated by the C1 and by the grand occipital nerve [30–33]; the rectus capitis posterior of sub-occipitalis is innervated by the ophthalmic division of the trigeminal nerve and by the grand occipital nerve [34, 35]. In fact, temporalis and sub-occipitalis are more related to the trigemino-cervical complex (C1-C3) than levator scapulae (C3-C5) or middle scalene (C3-C8) and trapezius (C2-C4). Therefore, temporalis and sub-occipitalis are also the muscles in the trigemino-cervical complex more assessed and treated in migraine [7, 11, 19, 23]: different pharmacological and no-pharmacological treatments such as onabotulinumtoxin injections [36], nerve stimulation or block [32–34], and manual therapy [7, 23, 37–39] target these muscles.

This study was limited by the absence of gender and age stratification. Gender and age play a role in pain perception, gender may play a role also in muscle morphology [40], but the sample size did not allow to stratify the population. Nevertheless, the sample was homogenous in terms of gender and age. On the other hand, the present study provides the first comprehensive assessment of PPT in different muscles over trigeminal and extra-trigeminal area according to the Andersen guideline [19] that may clarify pain mechanisms and may provide practical implications.

## Conclusion

People with chronic and episodic migraine presented lower PPT than healthy controls both in the trigeminal and in the extra-trigeminal area. People with chronic migraine presented lower PPT than episodic migraine only in the trigeminal area. Temporalis and sub-occipitalis are the most sensitive muscles in people with chronic and episodic migraine. Even though the chronicity could lead to central sensitization and in a larger pain extent area, people with chronic migraine present more local hyperalgesia in the trigemino-cervical complex rather than widespread hyperalgesia. Therefore, pharmacological and non-pharmacological approaches, such as pharmacological injections, neuromodal stimulation, and physiotherapy, should target this area. Further research is required to establish the gender and age role in local and widespread hyperalgesia and the change in PPT in relation to different treatments.

## Data Availability

The data associated to this study can be requested to the corresponding author upon reasonable request.

## Abbreviations

*PPT*: Pressure pain threshold
*ANOVA*: Analysis of variance
*TFL*: Tensor fasciae lata
